# Inhibition of ovine *in vitro* fertilization by anti-Prt antibody: hypothetical model for Prt/ZP interaction

**DOI:** 10.1186/1477-7827-11-25

**Published:** 2013-03-26

**Authors:** Jorge Pimenta, João Sardinha, Carla C Marques, Ana Domingos, Maria C Baptista, João P Barbas, Ivo C Martins, Patrícia Mesquita, Pedro Pessa, Rui Soares, Aldino Viegas, Eurico Cabrita, EM António Horta, Carlos A Fontes, AM José Prates, MLN Rosa Pereira

**Affiliations:** 1 Unidade de Biotecnologia e Recursos Genéticos, Instituto Nacional de Investigação Agrária e Veterinária Santarém, Quinta da Fonte Boa, Vale de Santarém, 2005-048, Portugal; 2 CIISA, Faculdade de Medicina Veterinária (FMV), Universidade Técnica de Lisboa, Lisbon, Portugal; 3 REQUIMTE/CQFB, Departamento de Química, Faculdade de Ciências e Tecnologia, Universidade Nova de Lisboa, Caparica, Portugal; 4 IHMT-CMDT – Instituto de Higiene e Medicina Tropical, Centro de Malária e Doenças Tropicais, Lisbon, Portugal; 5 Instituto de Medicina Molecular, Faculdade de Medicina da Universidade de Lisboa, Av. Prof. Egas Moniz, Lisbon, 1649-028, Portugal; 6 Hospital Universitário de Coimbra, Coimbra, Portugal; 7 Escola Universitária Vasco da Gama, Coimbra, Portugal

**Keywords:** Prion proteins, Prt, Zona pellucida, Circular dichroism, Docking, Ovine, Reproduction

## Abstract

**Background:**

The impact of prion proteins in the rules that dictate biological reproduction is still poorly understood. Likewise, the role of *prnt* gene, encoding the prion-like protein testis specific (Prt), in ram reproductive physiology remains largely unknown. In this study, we assessed the effect of Prt in ovine fertilization by using an anti-Prt antibody (APPA) in fertilization medium incubated with spermatozoa and oocytes. Moreover, a computational model was constructed to infer how the results obtained could be related to a hypothetical role for Prt in sperm-zona pellucida (ZP) binding.

**Methods:**

Mature ovine oocytes were transferred to fertilization medium alone (control) or supplemented with APPA, or pre-immune serum (CSerum). Oocytes were inseminated with ovine spermatozoa and after 18 h, presumptive zygotes (n = 142) were fixed to evaluate fertilization rates or transferred (n = 374) for embryo culture until D6-7. Predicted ovine Prt tertiary structure was compared with data obtained by circular dichroism spectroscopy (CD) and a protein-protein computational docking model was estimated for a hypothetical Prt/ZP interaction.

**Results:**

The fertilizing rate was lower (P = 0.006) in APPA group (46.0+/−6.79%) when compared to control (78.5+/−7.47%) and CSerum (64.5+/−6.65%) groups. In addition, the cleavage rate was higher (P < 0.0001) in control (44.1+/−4.15%) than in APPA group (19.7+/−4.22%). Prt CD spectroscopy showed a 22% alpha-helical structure in 30% (m/v) aqueous trifluoroethanol (TFE) and 17% alpha in 0.6% (m/v) TFE. The predominant alpha-helical secondary structure detected correlates with the predicted three dimensional structure for ovine Prt, which was subsequently used to test Prt/ZP docking. Computational analyses predicted a favorable Prt-binding activity towards ZP domains.

**Conclusions:**

Our data indicates that the presence of APPA reduces the number of fertilized oocytes and of cleaved embryos. Moreover, the CD analysis data reinforces the predicted ovine Prt trend towards an alpha-helical structure. Predicted protein-protein docking suggests a possible interaction between Prt and ZP, thus supporting an important role for Prt in ovine fertilization.

## Background

The prion protein gene *prnp*, which encodes the protein PrP^Sc^, is the major structural component of prions, the infectious pathogens causing a number of disorders in mammals, including scrapie in sheep and bovine spongiform encephalopathy [1]. Moreover, the expression of *prnp* is also found in the gonads (testis and ovary) [2,3], indicating a role in germ cell differentiation during mammalian spermatogenesis [3].

A novel possibility for the function of prion proteins has emerged from the identification of the paralogue *prnd*, that lies downstream of *prnp* and encodes the Doppel (Dpl) protein [1]. *prnd* expression focuses on testis tissue at the adult, and takes an important role in maintaining sperm integrity, normal fertility and motion ability [1,4] and eventually in the sperm-oocyte interaction [5], which might be linked to its physiological role in acrosome biogenesis [6].

A third member of the prion gene family, *prnt* (that encodes the Prt protein), was later described as being closer to *prnd* than *prnp* in the human genomic sequence [7,8]. *prnd* and *prnt* genes are located in close proximity and in opposite orientations, a structural arrangement frequently encountered in genomic regions that have a similar structural organization and expression profiles [7]. All three isoforms of human *prnt* are exclusively expressed in the adult testis and are not present in any of the foetal tissues, including testis [7]. In caprine, *prnt* is weakly and stochastically expressed in both testes and ovaries at various development stages, suggesting that the expression pattern of this gene differs between ruminant and human [9]. Recent results demonstrated that Prt is also found in the ram germinal cells [10]. Prt was found in seminiferous tubules, along with the developing stages of germinal cells, but not in the wall of the spermatogenic epithelium or in Sertoli cells. Primarily expression in the nuclei of spermatogonia and spermatocytes, and subsequently in the elongated spermatides and in the spz acrosome, unrelated to spz capacitation, indicate that ovine Prt may play an important role in ram spermatogenesis, throughout spermatogenic cell proliferation and sperm maturation, as well as in fertilization.

Vertebrate oocytes are surrounded by an extracellular matrix called the zona pellucida (ZP) in mammals [11-13]. The different ZP glycoproteins share an apparent overall similar architecture [14] and are classified in six gene subfamilies (reviewed in Goudet et al. [11] and Spargo et al. [15]) with the same nomenclature used interchangeably hereafter to describe the corresponding genes and proteins. In mammals, all ZPs share ZP2 and ZP3 proteins (and one or both of the ZP1 and ZP4 proteins), indicating that both genes have functional importance [11]. The ability of sperm to bind to ZP is one of the most important indicators for sperm fertilizing ability [16-20]. Therefore, and based in the Prt location and expression in male germ cells [7,9,10], it is reasonable to suggest a role for Prt during early sperm-oocyte binding events, that could be mediated by ZP proteins. The main aim of the present investigation is thus to explore the role of Prt in fertilization, given the potential impact that it may have in the reproduction of mammals, including humans. To accomplish this (for simplicity and given the host lab previous experience [10]), a ovine model system was employed, with two distinct yet complementary approaches: one focused on data collected *in vivo* (via classical cell culture assays), and the other zoomed in the role of individual molecules (via biophysical assays and *in silico* analysis of the proteins involved). In the first approach, ovine Prt was partly inhibited during fertilization by addition of an anti-Prt polyclonal antibody (APPA) to the culture medium. Fertilization and cleavage rates were determined. In the second approach, Prt predicted tertiary structure was refined and compared with data from CD spectroscopy. Then, a forecast computational model was undertaken for protein-protein docking between predicted tertiary structures of Prt and ZPs.

## Methods

Ovine semen collection and *in vitro* fertilization protocols [21,22] were reviewed by the Ethics Committee of CIISA/FMV and approved by the Animal Care Committee of the National Veterinary Authority (Direção-Geral de Alimentação e Veterinária, Portugal), following the appropriate European Union guidelines [23].

### Experimental design

In order to define a role for Prt in ovine fertilization, we hypothesized that the antiserum blockage of ram Prt would limit the fertilizing capacity of ram spermatozoa and subsequent embryo development. As Prt was detected (as described in Pimenta et al. [10]) in the sperm head apical ridge subdomain of ejaculated ram spz (corresponding to the acrosome region) and along *in vitro* capacitation, it seemed reasonable to investigate its connection to the first steps of sperm-zona binding. To test our hypotheses, different experiments were conceptualized:

### Does Prt blockage interfere with fertilization rates?

Mature ovine oocytes (n = 516) were randomly divided and transferred to fertilization medium alone (control group) or supplemented with 1 μLmL^-1^ (v/v) of anti-Prt serum (APPA group), or with 1 μLmL^-1^ (v/v) of pre-immune serum (CSerum group) and then co-cultured for 18 h with a mixed pool of frozen/thawed spermatozoa (from three Merino rams) previously submitted to swim up. Afterwards, samples of oocytes were fixed and stained for fertilization assessment (18 h, n = 142). Analysis of data representing 4 replicates of fertilization evaluation was performed.

### Does Prt blockage interfere with embryo production rates?

Presumptive zygotes (from previous steps, n = 374) were denuded and cultured until the stage of 2-4-8 cells embryos. After assessing cleavage, embryo development proceeded until the blastocyst stage, and D6-7 embryo rates were evaluated. Analysis of data representing 4 replicates of embryo production was performed.

### Can Prt bind to ZP proteins?

The predicted (I-TASSER software [24,25]) tridimensional structure of ovine Prt was refined (Amber software [26]), and compared to data from CD spectroscopy of Prt in 30% (m/v) and 0.6% (m/v) aqueous TFE. The predicted 3D structures of bovine ZPs (ZP2, ZP3 and ZP4) with homologous ovine fragments were also refined, and used to test for Prt/ZP computational docking, with the HADDOCK software [27].

### Antibodies

Unless stated, all reagents used were provided from Sigma-Aldrich (St. Louis, USA). Mouse anti-ovine Prt polyclonal antibody (APPA) which has been shown to be specific for ovine Prt (as described in Pimenta et al. [10]), was used in these studies. Briefly, five to six-week-old female BALB/c mice were injected intraperitoneally with 20 μg of synthetic ovine Prt peptide (obtained from CASLO Laboratory ApS, Denmark; GenBank: ABO86196.1 and emulsified in incomplete Freund’s adjuvant), and boosted monthly (four times) with an equal mass of the referred peptide. Antibody responses generated against ovine Prt were measured by ELISA, and Prt location in semen was demonstrated by Western Blot assay of a protein extract from ram spermatozoa. Membranes were incubated with APPA, with and without previous peptide blocking in order to demonstrate the antibody specificity. Also, mouse serum was collected prior to immunization (pre-immune), and used as a blank (CSerum).

### Semen collection and cryopreservation

Semen collection was conducted at the experimental farm of INIAV. Semen was collected from native Portuguese Merino rams, already identified by its good *in vivo* and *in vitro* fertility results, using an artificial vagina. Immediately after collection, each ejaculate was evaluated for volume, motility and concentration. Good quality ejaculates (mass motility >4; individual motility >60%; concentration >2.5 × 10^9^ spz mL^−1^) were diluted using an extender containing a solution of 45.0 g L^-1^ TRIS, 24.4 gL^-1^ citric acid, 5.6 g L^-1^ glucose, 15% egg yolk (v/v), 6.6% glycerol (v/v) and antibiotics. The diluted semen was loaded into 0.25 ml mini-straws (300 × 10^6^ spz), refrigerated till 4°C and frozen in nitrogen vapours as described in Valente et al. [21].

### *In vitro* fertilization (IVF)

Ovine ovaries collected at the local slaughterhouse were transported to the laboratory in Dulbecco’s phosphate buffer saline (PBS, GIBCO) at 37°C. PBS was supplemented with 0.15% (w/v) of bovine serum albumin (BSA, Fraction V) and 0.05 mg mL^−1^ of kanamycin. At the laboratory, the 2–6 mm follicles were aspirated to obtain cumulus–oocyte complexes. These complexes were incubated in maturation medium (TCM-199, 10 μg mL^-1^ FSH, 0.3 mM sodium pyruvate, 100 μM cysteamine, 10 ng mL^−1^ epidermal growth factor, 10 μg mL^−1^ estradiol and 10 μL mL^−1^ gentamicin, as described in Romão et al. [28]), at 38.5°C and 5% CO_2_ for 22 h. For oocyte fertilization, a pool of frozen/thawed semen from 3 Merino rams was used in each session. After thawing three straws, pooled sperm motility was immediately examined. Then spermatozoa were submitted to swim-up in capacitation medium (modified Bracket’s medium containing 20% ovine superovulated oestrus serum) at 38.5°C and 5% CO_2_ for 1 hour. The upper layer was centrifuged at 225 *g* for 5 minutes and the supernatant rejected, according to Pereira et al. [22]. The remaining pellet of spermatozoa was evaluated prior to be used to fertilize the oocytes in fertilization medium. Mature ovine oocytes were transferred to *in vitro* fertilization medium alone (control group) or supplemented with 1 μL mL^-1^ (v/v) of anti-Prt serum (APPA group), or with 1 μL mL^-1^ (v/v) of pre-immune serum (CSerum group) and then co-cultured with the spermatozoa (1 × 10^6^ spz mL^−1^) for 18 h. The fertilization medium consisted of synthetic oviductal fluid (SOF) containing glutamine (1.5 μg mL^-1^), BME (20 μL mL^-1^) and MEM amino acids (10 μL mL^-1^), gentamicin (10 μL mL^-1^) and 10% ovine superovulated oestrus serum. Samples of oocytes after 18 h of insemination were fixed and stained for fertilization assessment, while the remaining inseminated oocytes proceeded to assess embryo development.

### Fertilization assessment

Eighteen hours after oocyte insemination, samples of presumptive zygotes from all groups (control, n = 42; APPA n = 50, CSerum n = 50) were fixed and stained with 1% aceto-lacmoid. Fertilization was considered to occur by the observation in a phase microscope of either a decondensed sperm head or 2 pro-nuclei or zygotes and also when observing more than 2 swollen sperm heads or 2 pronuclei within a single oocyte. Fertilization rate was calculated as the number of fertilized oocytes per number of fertilized and matured oocytes. Immature and unidentified oocytes were not accounted.

### Embryo culture

After fertilization, presumptive zygotes were denuded and cultured in droplets of SOF enriched with amino acids (20 μL mL^−1^ BME, 10 μL mL^−1^ MEM and 6 mg mL^−1^ bovine serum albumin (BSA,) at 38.5°C, under 5% O_2_, 5% CO_2_ and 90% N_2_ in an humidified atmosphere until the stage of 2-4-8 cell embryos. After assessing cleavage, embryo development proceeded until the blastocyst stage in SOF plus BSA and 10% (v/v) fetal calf serum. Cleavage rate was calculated as the number of cleaved embryos per number of inseminated oocytes and D6-7 embryo rate as the number of morulae and blastocyts at these days per number of cleaved embryos.

### Sequence alignment

Sequence alignment was undertaken with the M-Coffee server, a web server that computes multiple sequence alignments (MSAs) by running several MSA methods and combining their output into one single model [29].

### Circular Dichroism Spectra

The secondary structure of the Prt peptide previously used to induce APPA antiserum production was studied in two aqueous TFE concentrations (0.6 and 30% v/v) by CD spectroscopy. CD measurements were executed on a Jasco 815 spectrometer between 190 and 260 nm, using of 0.5 cm of path-length cells. The spectra were measured at a peptide concentration of 15 μM m/v (in 30% v/v TFE) and 10.2 μM m/v (in 0.6% v/v TFE). The secondary structure content of Prt peptide was calculated using the CD spectrum deconvolution software CDNN [30] which calculates the secondary structure of this peptide by comparison with a database of CD spectra of known protein structures.

### Predicted Prt/ZP docking protocol

Since the complete ovine ZP sperm-binding proteins sequences are not known, the predicted (with I-TASSER software [24,25]) tertiary structures of bovine ZP sperm-binding protein sequences [ZP2 (UniProtKB: Q9BH10), ZP3(UniProtKB: P48830) and ZP4(UniProtKB: Q9BH11) [31-33]] were used, replacing the corresponding bovine sequence, with the homologous ovine fragments [ovine ZP2 (UniProtKB: E5FYD9), and ovine ZP3 (UniProtKB: E5FYG4) [31]].

The ovine Prt (UniProtKB: A4ULE2) and bovine ZPs (with the referred ovine fragments) protein structures predicted by I-TASSER were energy minimized by molecular mechanics in AMBER 12 [26] using the ff12SB force field through 1000 steps of the steepest descent method, followed by the conjugate gradient method until a 0.0001 convergence Kcal.mol^-1^ was achieved. The Prt peptide was refined afterwards in explicit solvent using an isometric truncated octahedron TIP3P-water box of 11 Å and the proper number of counter ions was added using tleap as implemented in AMBER package. The simulation was carried out using periodic boundary conditions following a five-step protocol: The first step consisted in a 20000 cycles of minimization to remove any possible unfavorable contacts between the water and the peptide. The first 50 × 10^2^ cycles of the minimization were performed with the steepest descent method, followed by the conjugate gradient method. In this step, the solute was restrained in the cartesian space using a harmonic potential restraint (weight 200 kcal mol^–1^.Å^–2^). Subsequently, a 10 × 10^3^ cycles of minimization (30 × 10^2^ steps of steepest descent and 7000 steps of conjugate gradient method) without restraints was performed. The system was then heated up to 298 K for 50 ps using a NVT ensemble and a weak positional restraint (10 mol^–1^Å^–2^) on the solute, to avoid wild structural fluctuations, using the Langevin thermostat with a collision frequency of 1 ps^-1^. The positional restraint was removed and a molecular dynamics run in an isothermal-isobaric (NPT) ensemble at 298 K for 1 ns was performed for equilibration at 1 atm with isotropic scaling and a relaxation time of 2 ps. Finally, NPT data production run was carried out for 10 ns and snapshots of the system were saved to a trajectory file every 0.2 ps. All bonds involving hydrogen atoms were constrained with the SHAKE algorithm [34] allowing the use of a 2 fs time step. The Particle Mesh Ewald method [35] was used to treat the long-range electrostatic interactions and the non-bonded van der Waals interactions were truncated with a 9 Å cut-off. The structural collected data were analyzed with the PTRAJ module as implemented in the AMBER package. The MD trajectory was clustered by RMSD similarity using the average-linkage clustering algorithm [36]. A representative conformation of the cluster with larger population was taken for further docking studies.

The docking simulations were performed with the HADDOCK webserver [37], using the structures previously optimized. The interface prediction in both partners (active and passive residues) were determined through the CPORT [38] prediction method that is optimized for use with HADDOCK. The HADDOCK docking protocol was performed as described elsewhere [39]. The first docking step consisted in a rigid body energy minimization. 10 × 10^3^ complex conformations were calculated at this stage. The 500 best solutions were then selected for further simulated annealing refinements. Firstly the two proteins were considered as rigid bodies and their respective orientation was optimized, then the side chains at the interface were allowed to move in a second simulated annealing simulation. In the third simulating annealing simulation both backbone and side chains at the interface were allowed to move. The resulting complex structures were energy minimized through 200 steps of the steepest descent method. In the final docking stage, the structures obtained were gently refined (100 MD heating steps at 100, 200 and 300 K followed by 750 sampling steps at 300 K and 500 MD cooling steps at 300, 200 and 100 K all with 2 fs time steps) in an 8 Å shell of TIP3P water molecules. A cluster analysis was performed on the final docking solutions using a minimum cluster size of 6. The root mean square deviation (RMSD) matrix was calculated over the backbone atoms of the interface residues using a 2.0 Å cut off. The resulting clusters were analyzed and ranked according to the HADDOCK score which consists in a weighted sum of intermolecular electrostatic, van de Waals, desolvation and AIR (ambiguous interaction restraints) energies, and a buried surface area term. All molecular diagrams were drawn with PyMOL 1.4.1. [40].

### Statistical analysis

The results were expressed as least squares means ± standard error. Data representing 4 replicates of fertilization stages evaluation and embryo production rates analysis were performed by the MIXED procedure of Statistical Analysis Systems Institute (SAS Inst., Inc., Cary, NC, USA). The mixed linear model included treatment (control, anti-Prt immune serum and pre-immune serum, in fertilization medium) as fixed effect and replicates as random effect. When significant effects were identified, values were compared using the T- or Bonferroni tests. Differences were considered significant when P ≤ 0.05.

## Results

### Fertilization and embryo rates

A total of 516 ovine mature oocytes were used to assess the fertilizing ability of ovine spermatozoa incubated in the presence and absence of anti-Prt serum and pre-immune serum. The anti-Prt serum clearly interfered (P = 0.006) with the fertilization rate (Table 1). This rate was lower in APPA group than in Control (P = 0.001) and CSerum (P = 0.05) groups. Accordingly the percentage of Metaphase II oocytes was higher in APPA group than in Control (P = 0.001) and Cserum (P = 0.05). CSerum group had more (P = 0.02) zygotes at synkaryosis stage than APPA group No differences (P > 0.05) were identified for sperm head decondensation; two pronuclei, embryo or polyspermic rates among groups (Table 1). In what concerns embryo production rates, only cleavage rates were affected (P < 0.001) by the presence of anti-Prt immune serum in the fertilization medium, being higher (P < 0.0001) in control (44.1 ± 4.15%) than in APPA (19.7 ± 4.22%) group. No differences were identified for D6-7 embryo rates among groups (Table 2).

**Table 1:** **Assessment of *in vitro *fertilization of ovine oocytes**

**Treatment**	**MII**	**Decond**	**2 pronuclei**	**Synkaryosis**	**2-4 cells**	**Polyspermy**	**Fertilization**
	**(n) %**	**(n) %**	**(n) %**	**(n) %**	**(n) %**	**(n) %**	**(n) %**
Control	(10) 21.5 ± 7.47^a^	(1) 1.9 ± 2.76	(20) 52.5 ± 7.78	(1) 2.4 ± 3.31 ^ab^	(4) 11.0 ± 4.1	(3) 9.6 ± 3.45	(29) 78.5 ± 7.47^a^
APPA	(27) 54.0 ± 6.79^b^	(1) 1.6 ± 2.51	(13) 29.5 ± 7.07	(0) 0.0 ± 3.01^b^	(3) 7.0 ± 3.7	(3) 8.0 ± 3.14	(20) 46.0 ± 6.79^b^
CSerum	(19) 35.5 ± 6.45^a^	(2) 3.5 ± 2.46	(20) 42.6 ± 6.92	(5) 10.2 ± 3.00^a^	(2) 4.7 ± 3.6	(1) 3.6 ± 3.07	(30) 64.5 ± 6.45^a^

Assessment of *in vitro* fertilization of ovine oocytes incubated in the absence (Control) and presence of anti-Prt serum (APPA) and pre-immune serum (Cserum) in 4 replicates. MII: metaphase II; Decond: sperm head decondensation; 2–4 cells: 2–4 cell embryos; Least squares mean values within columns with different letters differ statistically (P ≤ 0.05).

**Table 2:** Effect of anti-Prt serum on embryo and cleavage rates

**Treatment**	**Inseminated ** **oocytes (n)**	**Cleavage n (%)**	**D6/7 embryosn (%)**
Control	127	(63) 44.1 ± 4.15^a^	(9) 21.0 ± 5.12
APPA	118	(27) 19.7 ± 4.22^b^	(6) 24.3 ± 7.12
CSerum	129	(47) 32.2 ± 4.06^ab^	(8) 22.9 ± 5.56

Effect of spermatozoa incubation in the presence of anti-Prt serum (APPA) or pre-immune serum (CSerum) in the fertilization medium on embryo production rates in 4 replicates for cleavage and 3 replicates for D6/7 embryos (least squares means ± standard error). n: total number; data within columns with different superscripts are statistically different (P ≤ 0.05); Cleavage rate: number of cleaved embryos per number of inseminated oocytes; D6-7 embryo rate as the number of morulae and blastocyts at these days per number of cleaved embryos.

### CD Measurements

**Figure 1:**
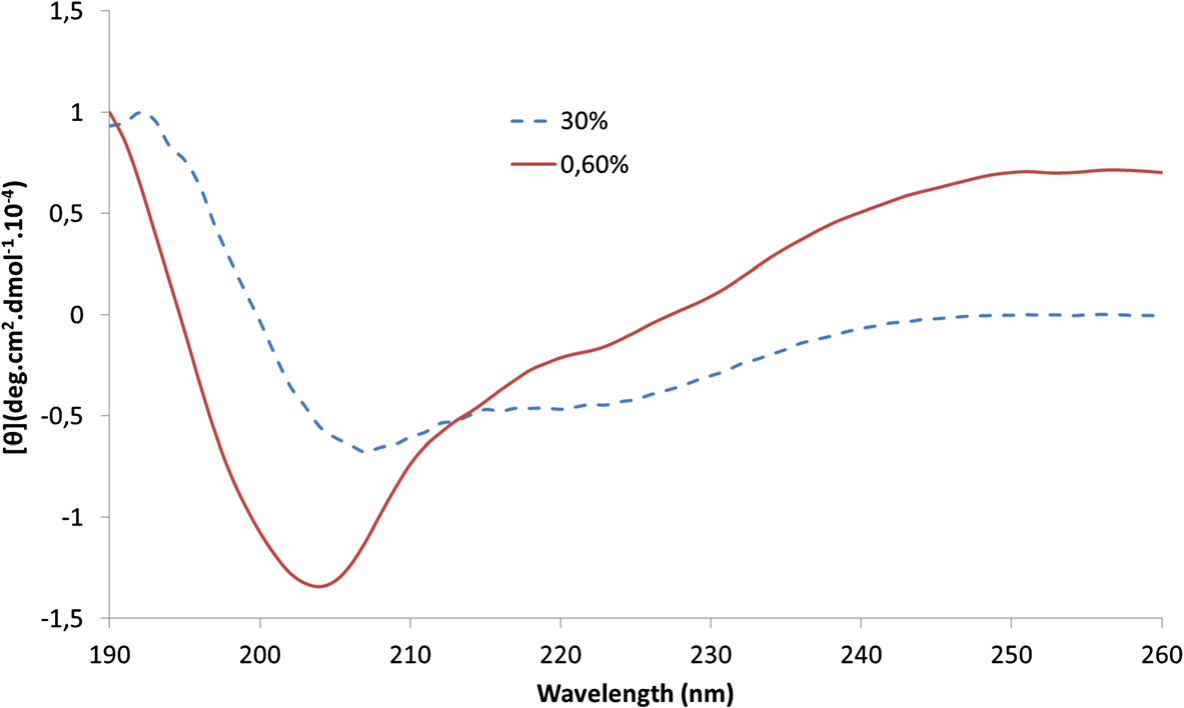
**CD spectrum of Prt peptide.** CD spectrum of Prt peptide in 0.6 (red line) and 30% (blue line) TFE. The final peptide concentration was 10.2 μM (0.6% TFE) and 15 μM (30% TFE). The temperature was 25°C. [*θ*], Molar elipticity.

Figure 1 shows the CD spectrum of Prt peptide dissolved in TFE. In the far UV region (180–240 nm), which corresponds to peptide bond absorption, CD spectrum exhibits two negative bands located at 204 (0.6% TFE) and 207 nm (30% TFE), combined with positives bands near 190 nm, suggesting the presence of a stable α-helical conformation over the TFE range. This is confirmed by secondary structure analysis using the CDNN [30] software that suggests that 17.6% and 22% of the Prt peptide populates an α-helical conformation (in 0,6 and 30% TFE, respectively). In aqueous solution it is mostly a random coil (data not shown).

Biophysical studies of aggregation processes frequently employ chemical cosolvents to reduce experimental variability, simulate cellular conditions, or induce the formation of atypical aggregate structures. The TFE fluorinated alcohol (2,2,2-trifluoroethanol), is one of the most commonly used membrane-mimicking cosolvents in these studies assays [41,42]. TFE tends to enhance the stability of backbone hydrogen bonds of which the carbonyl groups are from non-polar residues [43]. TFE has been shown to induce and stabilize α-helices in sequences with intrinsic helical propensity [44] without altering helix limits [45] and is a solvent considered to mimic the hydrophobic solvent-excluded environment, which may form early in the protein folding pathway [46]. Moreover, Lehrman et al. [47] suggested that the TFE-enhanced α-helicity, is indicative of the α-helical propensity.

### Sequence alignment between ZP domains

**Figure 2:**
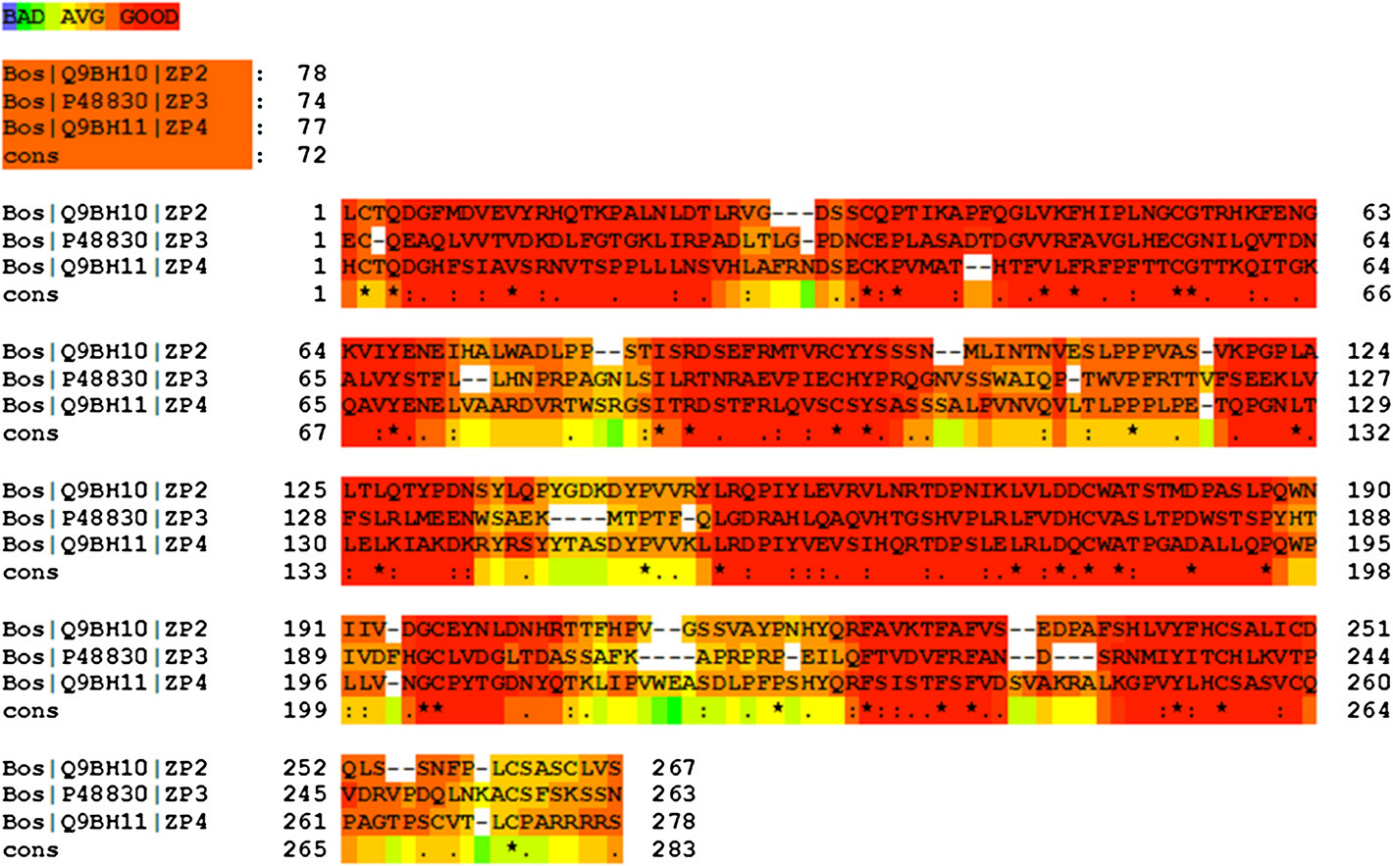
**Sequence alignment of bovine Zona pellucida sperm-binding protein ZP domains.** Top lane: Bovine ZP2 (UniProtKB: Q9BH10) ZP domain sequence. Second lane: Bovine ZP3 (UniProtKB: P48830) ZP domain sequence. Third lane: Bovine ZP4 (UniProtKB: Q9BH11) ZP domain sequence. Fourth lane: consistency. Asterisk (*) denotes identical residue; colon (:), conservative change; period (.), related substitution. Dark red indicates residues aligned in a similar fashion among all the individual MSAs (multiple sequence alignments). Dark yellow, orange and red residues can be considered to be reliably aligned. The top of the output provides the average consistency (ranging from of 0 to 100) for each sequence, indicating sequence alignment reliability (score lower than 50 is considered poor). Hereafter, amino acids are represented by single letter code for amino acid residues. The alignment was performed using the M-Coffee program [29].

In this colored output (Figure 2), each residue has a color that indicates the agreement of the individual multiple sequence alignments (MSAs) with respect to the alignment of that specific residue. Residues in red are in perfect agreement with every constituting multiple alignments. Previous analysis indicates that 90% of the residues having an individual score of 7 or higher (dark yellow, orange and red) are correctly aligned [29,48]. Average consistency presents a main score above 70 (Figure 2).

### Predicted Prt/ZP docking

Predicted Prt and ZP3-dimensional protein structures were obtained with the I-TASSER software [24] from amino-acid sequences, using mainly the crystal structures of the full-length chicken sperm receptor ZP3 (PDB: 3NK3 and 3NK4) as templates for bovine ZP2, ZP3 and ZP4 homology modeling, and *ab initio* structure modeling in the case of ovine Prt, as described in Wu et al. [49]. Compared with other modeling methods, the average performance of I-TASSER is either much better or is similar within a lower computational time [49], and models exhibiting higher C-scores (a confidence score for estimating the quality of predicted models by I-TASSER), were further optimized with the Amber software [26]. The biomolecular modeling program HADDOCK [37,39] was then used to generate a structural model of the possible interaction between Prt and ZPs. As can be seen from the intermolecular energies in Table 3, the associations are dominated by electrostatic interactions.

**Table 3:** Structural statistics

	**ZP2-Prt**	**ZP3-Prt**	**ZP4-Prt**
HADDOCK score	−49.5 ± 10.1	−61.4 ± 12.7	−33.3 ± 2.6
RMSD from the overall lowest­energy structure	0.8 ± 0.5	0.6 ± 0.3	2.8 ± 0.4
E_vdw_ (kcal mol^-1^)	−97.2 ± 8.6	−116.1 ± 7.7	−116.8 ± 19.8
E_elec_ (kcal mol^-1^)	−471.9 ± 47.7	−315.7 ± 26.5	−220.4 ± 40.3
Buried surface area (Å^2^)	3310.9 ± 94.0	2998.5 ± 63.3	2892.0 ± 241.2

Structural statistics obtained from the 10 best structures of each ZP-Prt cluster using HADDOCK [37,39].

**Figure 3:**
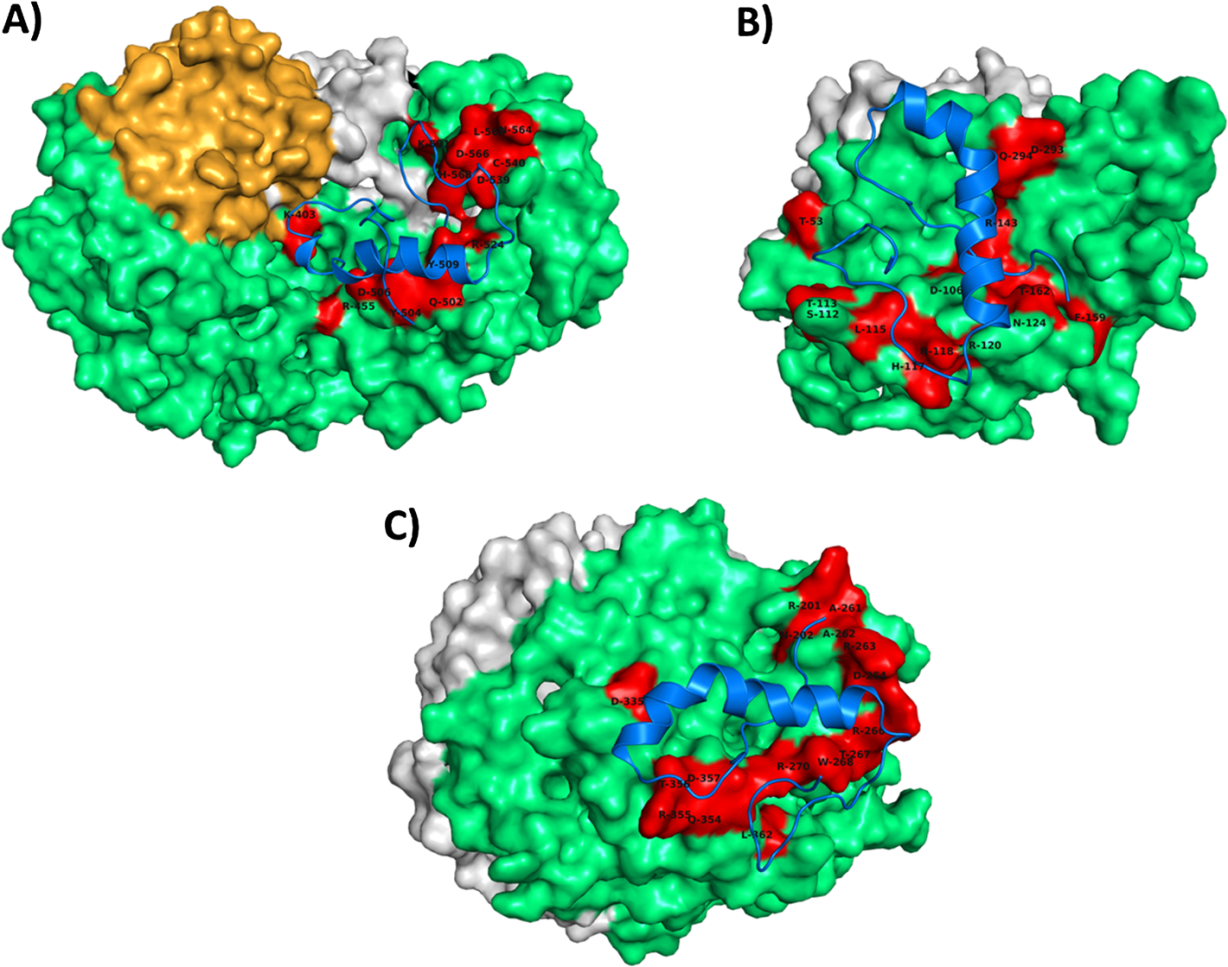
**Prt/ZP predicted binding.** (**A**) Prt/ZP2 model; (**B**) Prt/ZP3 model; (**C**) Prt/ZP4 model. ZPs and Prt are shown respectively in surface and cartoon representations. Residues 36–150 of bovine ZP2 are colored in orange. ZP2 (36–635), ZP3 (23–346) and ZP4 (18–461) chains, are colored in light green. Polar contacts are colored in red (corresponding ZP aminoacids are indicated with one-letter code). N-terminal signal peptide, and C-terminal propeptide (both removed in mature form), are colored in light gray. Ovine Prt model (cartoon) is colored in blue. Based on UniProtKB/Swiss-Prot sequences [Prt (A4ULE2); ZP2 (Q9BH10); ZP3(P48830); ZP4(Q9BH11)].

**Figure 4:**
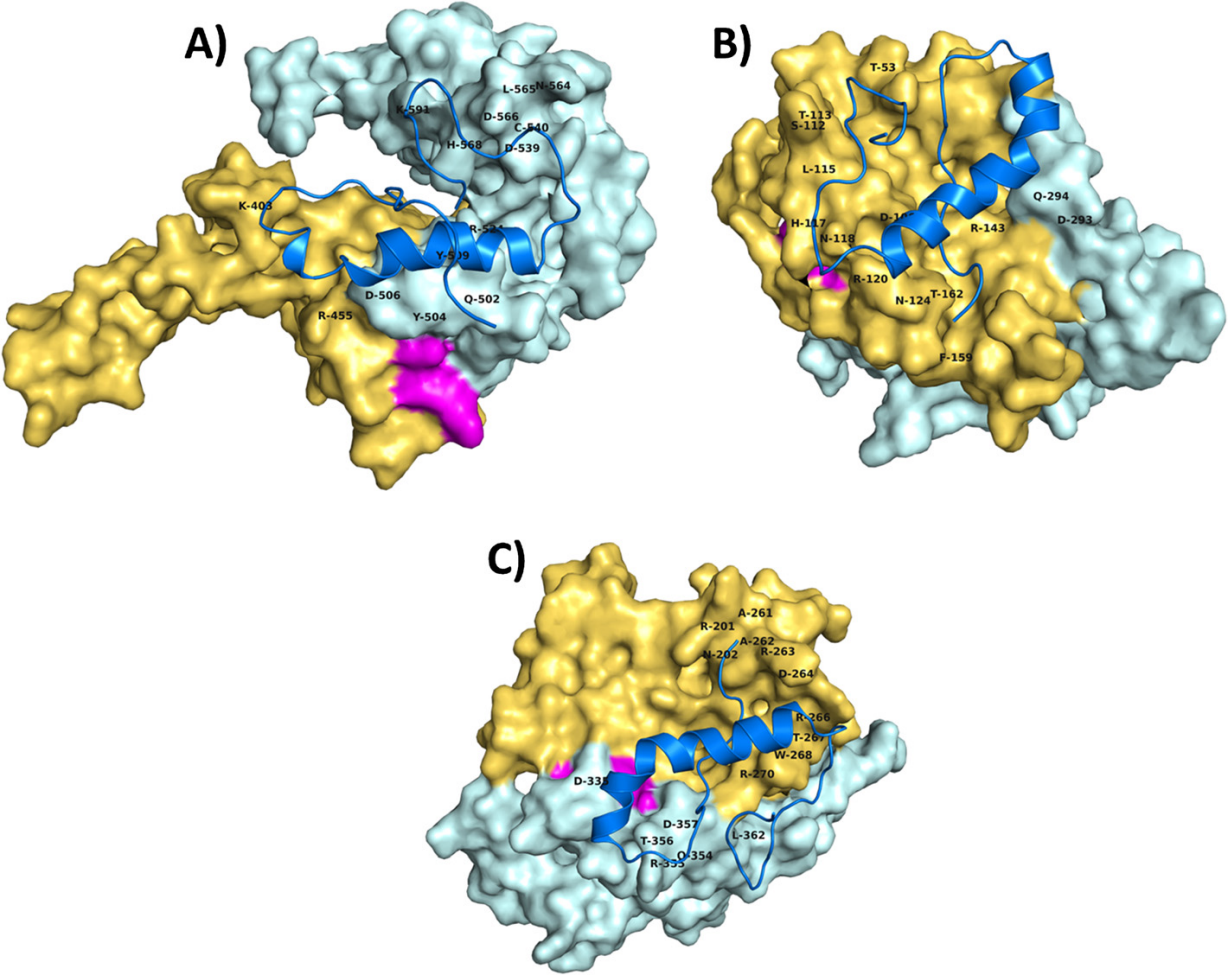
**Prt/ZP domain, predicted binding.** (**A**) Prt/ZP2 (ZP domain) model; (**B**) Prt/ZP3(ZP domain) model; (**C**) Prt/ZP4(ZP domain) model. ZP domains and Prt are shown respectively in surface and cartoon representations. N-terminal region of ZP domain is coloured in yellow-orange. C-terminal region of ZP domain is coloured in pale cyan. Expected Internal Hydrophobic Patch (IHP) interdomain linker (using the mouse and human IHP linkers as templates; as described in Jovine et al. [67]), are coloured in magenta. Polar contacts with corresponding ZP amino acids are indicated with one-letter code. Based on UniProtKB/Swiss-Prot sequences [Prt (A4ULE2); ZP2 (Q9BH10; a.a. 366–632); ZP3(P48830; a.a. 44–306); ZP4(Q9BH11; a.a. 188–465)].

Clusters with the lowest not higher scores were selected and the polar contacts mapped, thus giving structural insights of ZP-Prt interaction, as represented in Figures 3 and 4. Images of the three best presenting clusters (Haddock scores), for ZP2, ZP3 and ZP4 are also available as additional material files (Additional file 1: Figure S1). According to the predicted docking results, Prt seems to exhibit a trend towards the ZP domain within the ZP protein chains, that are structural elements predicted to exist as a bipartite structure corresponding to ZP-N and ZP-C regions tethered by a linker [50]. In the case of ZP3, polar contacts are predominantly distributed along the expected bovine ZP-N subdomain (Figure 4). As such, and in order to test if the ZP-N of mouse sperm receptor ZP3, could provide a better template for the homologous bovine subdomain, we specified mouse ZP-N (ZP3) crystal form I (PDB: 3D4C) as a template for bovine ZP-N (ZP3) 3D modeling with I-TASSER. Interestingly, the best C-score was again obtained using the full-length chicken sperm receptor ZP3 as a template (not shown), reinforcing the attained models.

## Discussion

In our previous study [10] we determined the location of ovine *prnt* along the developing stages of ram germinal cells and specifically at the sperm head apical ridge subdomain of ejaculated and capacitated ovine spermatozoa. Therefore, the aim of our present work was to determine if Prt, like Doppel [5,51], could also play a role in the fertilization process. In order to elucidate the molecular foundations of fertilization, we employed an antibody based approach by which we blocked Prt function via an anti-Prt antibody, following previous similar approaches by other authors [52]. Herein we showed that adding APPA, an antibody that binds and subsequently blocks Prt function, to a culture medium where the spermatozoa are actively attaching, penetrating and fertilizing the oocytes, effectively and significantly (P = 0.006) decreases the fertilization rate, with the overall fertilizing ability of ram spermatozoa being lower than in control (fertilization rates: 46 ± 6.8 vs. 78.5 ± 7.5%, P = 0.001, Table 1). This seems to indicate a key role for Prt during fertilization. The cleavage rate of APPA group, which is as an indicator of the progress of fertilization was also lower than in control (19.7 ± 4.2 vs. 44.1 ± 4.2%, P < 0.0001; Table 2), although not significantly different from C Serum group. Interestingly, no differences were identified for D6-7 embryo rates among groups, implying that no repercussions of APPA blockage on the development from 2–4 cells embryos to blastocyst were identified. Taken together, these observations suggest that Prt could exert its main function in the fertilization process, potentially in the initial steps due to its location in the acrosome of ovine spermatozoa.

Ram Prt is readily detected in germ cells (but not in testis somatic cells) throughout spermatogenesis and spermiogenesis (the final stage of spermatogenesis, that consists of the complex differentiation of spermatids into spermatozoa), on fresh sperm and along the capacitation process [10].

In the case of the other prion family proteins, Dpl was detected both in germ and somatic Leydig, Sertoli and epididymal epithelial cells [6,9,53-56], and PrP^C^ was detected in germ and somatic epididymal epithelial cells [3,56-60]. Finally, the protein Shadoo (Sho), a paralogue of prion protein [61], also reveals testis expression but only in specific somatic cell types of the testicle (namely interstitial Leydig cells) [62].

From the outlined data, it is possible to observe that the prion family genes (*prnp*, *prnd*, *sprn* and *prnt*) exhibit different expression patterns in mammalian testis, so it seems conceivable that they might also present gentle deviations in terms of their function in reproduction, as has been previously suggested by Makrinou et al. [7]. Thus, it appears reasonable to theorize that Prt could be involved in the early steps of fertilization, along the sperm-zona binding process. A hypothesis-driven tridimensional computational prediction was therefore developed to help understand if the study of Prt/ZP binding could represent an interesting path that might be followed in future research. For that purpose, the predicted 3D structure (I-TASSER; [24,25]) of ovine Prt was refined with the Amber software [26] and compared with data obtained from CD spectroscopy (Figure 1) which confirmed the α-helical structure. Prediction of bovine ZPs (ZP2, ZP3 and ZP4) three-dimensional structures was also undertaken with the I-TASSER software.

In the present report we predicted a preferential binding ability between Prt and ZP domains (Figures 3 and 4). Computational modelling predicts a preferential docking for Prt within the ZP2 domain (residues 366–632), predominantly in its C-terminal region, downstream of the interdomain linker IHP (Internal Hydrophobic Patch; residues 485–491) and without any residues located inside the homologous ZP2 N-terminus domain (corresponding to bovine residues 36–150) described by Baibakov et al. [63] at the humanized zona pellucida. With respect to Prt/ZP3 binding, all predicted polar contacts are also located in the ZP domain (residues 44–306), with >85% of polar contacts inside the ZP-N subdomain, upstream of the predicted bovine IHP (residues 166–172). Also, predicted polar contacts for Prt/ZP4 binding seem to locate predominantly inside the N-terminal region of the ZP domain.

Preliminary findings and predicted analysis emphasize the role of ‘ZP domain’ modules, along with Prt, at the early steps of mammalian fertilization. ZP domains are structural elements, found in hundreds of extracellular proteins having diverse functions [64,65], and from a wide variety of organisms, from nematodes to mammals, consisting of ∼ 260 aminoacids not amino acids positioned close to the C-terminus of the polypeptide [14]. This structural element characterized by a set of eight highly conserved cysteines, is predicted to exist as a bipartite structure corresponding to ZP-N and ZP-C regions tethered by a linker [50]. Recent in vivo analyses indicate that the functional organization of ZP-N is conserved throughout species, suggesting also that alteration of ZP-N structure affects polymerization of ZP-domain proteins [66]. Also, the conserved duplicated motif that include the IHP linker located between ZP-N and ZP-C domains, and the external hydrophobic patch (EHP; an integral part of the ZP-C domain according to Lin et al., assume an important role in the assembly of mouse ZP proteins [67]. Moreover, preliminary findings suggest that ZP domain (aa residues 273–551) of human ZP1 is sufficient to induce an acrosome reaction [68].

Data presented in this manuscript further highlight the importance of Zp domains and prion-like proteins in reproduction, requesting further studies so as to unearth the motifs and molecular mechanisms involved in the fertilization process, hoping that with such background information, results may then be interpreted in a more reliable manner.

## Conclusions

Our results point out a role for spermatozoa Prt in ovine fertilization, possibly through a structural interaction with ZP proteins. Therefore, the search for sperm surface proteins that function in sperm-zona binding, should consider Prt as a potential candidate that may help to elucidate the underlying mechanisms behind conception. Further studies of this Prt prion-like protein function could lead to a better understanding of the ovine fertilization process, eventually providing avenues into the clinical treatment of infertility or towards the development of new contraceptive approaches that could potentially include sperm-zona blockage.

## Abbreviations

APPA: Anti-ovine Prt polyclonal antibody; CD: Circular dichroism spectroscopy; CSerum: Pre-immune serum; Dpl: Doppel protein; IHP: Internal Hydrophobic Patch; MSAs: Multiple sequence alignments; prnd: Prion-like protein doppel gene; prnp: Prion protein gene; prnt: Prion protein testis-specific gene; PrP: Prion protein; PrPC: Cellular isoform of PrP; PrPSc: Scrapie isoform of PrP; Prt: Prion protein testis specific; Sho: Shadoo protein; sprn: Shadow of prion protein gene; spz: Spermatozoa; TFE: 2,2,2-trifluoroethanol; TSE: Transmissible spongiform encephalopathies; ZP: Zona pellucida

## Competing interests

The authors declare that they have no competing interests.

## Authors’ contributions

JP and RMLNP conceived and designed the experiments. All authors have been involved in acquisition of data, contributed to the writing and editing of this manuscript and read and approved the final manuscript.

## Supplementary Material

Additional file 1 **Figure S1.**Images of the three best presenting clusters (Haddock scores), for (A) ZP2, (B) ZP3 and (C) ZP4. Click here for file

## References

[B1] Mastrangelo P Westaway D The prion gene complex encoding PrP(C) and Doppel: insights from mutational analysis Gene 2001 275 1 18 10.1016/S0378-1119(01)00627-8 11574147

[B2] Bendheim PE Brown HR Rudelli RD Scala LJ Goller NL Wen GY Kascsak RJ Cashman NR Bolton DC Nearly ubiquitous tissue distribution of the scrapie agent precursor protein Neurology 1992 42 149 156 10.1212/WNL.42.1.149 1346470

[B3] Fujisawa M Kanai Y Nam SY Maeda S Nakamuta N Kano K Kurohmaru M Hayashi Y Expression of Prnp mRNA (prion protein gene) in mouse spermatogenic cells J Reprod Dev 2004 50 565 570 10.1262/jrd.50.565 15514463

[B4] Guan F Shi G Pan L Liu N Liu S Yang L [Doppel protein and its effects on animal reproduction] Sheng Wu Gong Cheng Xue Bao 2009 25 170 175 19459319

[B5] Behrens A Genoud N Naumann H Rulicke T Janett F Heppner FL Ledermann B Aguzzi A Absence of the prion protein homologue Doppel causes male sterility EMBO J 2002 21 3652 3658 10.1093/emboj/cdf386 12110578 PMC125402

[B6] Serres C Peoc'h K Courtot AM Lesaffre C Jouannet P Laplanche JL Spatio-developmental distribution of the prion-like protein doppel in Mammalian testis: a comparative analysis focusing on its presence in the acrosome of spermatids Biol Reprod 2006 74 816 823 10.1095/biolreprod.105.047829 16421231

[B7] Makrinou E Collinge J Antoniou M Genomic characterization of the human prion protein (PrP) gene locus Mamm Genome 2002 13 696 703 10.1007/s00335-002-3043-0 12514748

[B8] Harrison PM Khachane A Kumar M Genomic assessment of the evolution of the prion protein gene family in vertebrates Genomics 2010 95 268 277 10.1016/j.ygeno.2010.02.008 20206252

[B9] Kocer A Gallozzi M Renault L Tilly G Pinheiro I Le Provost F Pailhoux E Vilotte JL Goat PRND expression pattern suggests its involvement in early sex differentiation Dev Dyn 2007 236 836 842 10.1002/dvdy.21066 17226816

[B10] Pimenta J Domingos A Santos P Marques CC Cantante C Santos A Barbas JP Baptista MC Horta AE Viegas A Is prnt a pseudogene? Identification of ram Prt in testis and ejaculated spermatozoa PLoS One 2012 7 e42957 10.1371/journal.pone.0042957 22937002 PMC3427297

[B11] Goudet G Mugnier S Callebaut I Monget P Phylogenetic analysis and identification of pseudogenes reveal a progressive loss of zona pellucida genes during evolution of vertebrates Biol Reprod 2008 78 796 806 10.1095/biolreprod.107.064568 18046012

[B12] Monne M Jovine L A structural view of egg coat architecture and function in fertilization Biol Reprod 2011 85 661 669 10.1095/biolreprod.111.092098 21715714

[B13] Bogliolo L Ledda S Innocenzi P Ariu F Bebbere D Rosati I Leoni GG Piccinini M Raman microspectroscopy as a non-invasive tool to assess the vitrification-induced changes of ovine oocyte zona pellucida Cryobiology 2012 64 267 272 10.1016/j.cryobiol.2012.02.010 22387147

[B14] Jovine L Darie CC Litscher ES Wassarman PM Zona pellucida domain proteins Annu Rev Biochem 2005 74 83 114 10.1146/annurev.biochem.74.082803.133039 15952882

[B15] Spargo SC Hope RM Evolution and nomenclature of the zona pellucida gene family Biol Reprod 2003 68 358 362 12533396 10.1095/biolreprod.102.008086

[B16] Liu DY Baker HW Tests of human sperm function and fertilization in vitro Fertil Steril 1992 58 465 483 1521638 10.1016/s0015-0282(16)55247-9

[B17] Franken DR Kruger TF Oehninger S Coddington CC Lombard C Smith K Hodgen GD The ability of the hemizona assay to predict human fertilization in different and consecutive in-vitro fertilization cycles Hum Reprod 1993 8 1240 1244 8408521 10.1093/oxfordjournals.humrep.a138234

[B18] Oehninger S Mahony M Ozgur K Kolm P Kruger T Franken D Clinical significance of human sperm-zona pellucida binding Fertil Steril 1997 67 1121 1127 10.1016/S0015-0282(97)81449-5 9176454

[B19] Barratt CL Publicover SJ Interaction between sperm and zona pellucida in male fertility Lancet 2001 358 1660 1662 11728536 10.1016/S0140-6736(01)06727-7

[B20] Liu DY Garrett C Baker HW Clinical application of sperm-oocyte interaction tests in in vitro fertilization–embryo transfer and intracytoplasmic sperm injection programs Fertil Steril 2004 82 1251 1263 10.1016/j.fertnstert.2003.10.057 15533339

[B21] Valente SS Pereira RM Baptista MC Marques CC Vasques MI Pereira MV Horta AE Barbas JP In vitro and in vivo fertility of ram semen cryopreserved in different extenders Anim Reprod Sci 2010 117 74 77 10.1016/j.anireprosci.2009.04.007 19482446

[B22] Pereira RM Mesquita P Batista M Baptista MC Barbas JP Pimenta J Santos IC Marques MR Vasques MI Silva Pereira M Doppel gene polymorphisms in Portuguese sheep breeds: insights on ram fertility Anim Reprod Sci 2009 114 157 166 10.1016/j.anireprosci.2008.10.003 19028030

[B23] Council Directive 86/609/EEC On the approximation of laws, regulations and administrative provisions of the Member States regarding the protection of animals used for experimental and other scientific purposes Offical Journal of the European Communities 1986 L358 1 29

[B24] Roy A Kucukural A Zhang Y I-TASSER: a unified platform for automated protein structure and function prediction Nat Protoc 2010 5 725 738 10.1038/nprot.2010.5 20360767 PMC2849174

[B25] Zhang Y Template-based modeling and free modeling by I-TASSER in CASP7 Proteins 2007 69 Suppl 8 108 117 17894355 10.1002/prot.21702

[B26] Case DA Darden TA Cheatham TE Simmerling CL Wang J Duke RE Luo R Walker RC Zhang W Merz KM Amber 12 2012 San Francisco: University of California

[B27] de Vries SJ van Dijk M Bonvin AMJJ The HADDOCK web server for data-driven biomolecular docking Nat Protocols 2010 5 883 897 10.1038/nprot.2010.32 20431534

[B28] Romão R Marques CC Baptista MC Vasques MI Barbas JP Horta AEM Carolino N Bettencourt E Plancha C Rodrigues P Pereira RM Evaluation of two methods of in vitro production of ovine embryos using fresh or cryopreserved semen Small Ruminant Research 2013 110 36 41 10.1016/j.smallrumres.2012.07.029

[B29] Moretti S Armougom F Wallace IM Higgins DG Jongeneel CV Notredame C The M-Coffee web server: a meta-method for computing multiple sequence alignments by combining alternative alignment methods Nucleic Acids Res 2007 35 W645 648 10.1093/nar/gkm333 17526519 PMC1933118

[B30] Bohm G Muhr R Jaenicke R Quantitative-analysis of protein far uv circular-dichroism spectra by neural networks Protein Eng 1992 5 191 195 10.1093/protein/5.3.191 1409538

[B31] Chen S Costa V Beja-Pereira A Evolutionary patterns of two major reproduction candidate genes (Zp2 and Zp3) reveal no contribution to reproductive isolation between bovine species BMC Evol Biol 2011 11 24 10.1186/1471-2148-11-24 21266067 PMC3037879

[B32] Ikeda K Yonezawa N Naoi K Katsumata T Hamano S Nakano M Localization of N-linked carbohydrate chains in glycoprotein ZPA of the bovine egg zona pellucida Eur J Biochem 2002 269 4257 4266 10.1046/j.1432-1033.2002.03111.x 12199704

[B33] Yonezawa N Fukui N Kuno M Shinoda M Goko S Mitsui S Nakano M Molecular cloning of bovine zona pellucida glycoproteins ZPA and ZPB and analysis for sperm-binding component of the zona Eur J Biochem 2001 268 3587 3594 10.1046/j.1432-1327.2001.02269.x 11422390

[B34] Ryckaert J-P Ciccotti G Berendsen HJC Numerical integration of the cartesian equations of motion of a system with constraints: molecular dynamics of n-alkanes J Comput Phys 1977 23 327 341 10.1016/0021-9991(77)90098-5

[B35] Darden T York D Pedersen L Particle mesh Ewald: An N [center-dot] log(N) method for Ewald sums in large systems J Chem Phys 1993 98 10089 10092 10.1063/1.464397

[B36] Shao J Tanner SW Thompson N Cheatham TE Clustering Molecular Dynamics Trajectories: 1, Characterizing the Performance of Different Clustering Algorithms Journal of Chemical Theory and Computation 2007 3 2312 2334 10.1021/ct700119m 26636222

[B37] De Vries SJ van Dijk ADJ Krzeminski M van Dijk M Thureau A Hsu V Wassenaar T Bonvin AMJJ HADDOCK versus HADDOCK: new features and performance of HADDOCK2.0 on the CAPRI targets Proteins-Structure Function and Bioinformatics 2007 69 726 733 10.1002/prot.21723 17803234

[B38] de Vries SJ Bonvin AMJJ CPORT: A Consensus Interface Predictor and Its Performance in Prediction-Driven Docking with HADDOCK PLoS One 2011 6 e17695 10.1371/journal.pone.0017695 21464987 PMC3064578

[B39] Dominguez C Boelens R Bonvin A HADDOCK: a protein-protein docking approach based on biochemical or biophysical information J Am Chem Soc 2003 125 1731 1737 10.1021/ja026939x 12580598

[B40] Schrödinger LLC The PyMOL Molecular Graphics System 2010 141

[B41] Otzen DE Amyloid formation in surfactants and alcohols: membrane mimetics or structural switchers? Curr Protein Pept Sci 2010 11 355 371 10.2174/138920310791330622 20423296

[B42] Anderson VL Webb WW A desolvation model for trifluoroethanol-induced aggregation of enhanced green fluorescent protein Biophys J 2012 102 897 906 10.1016/j.bpj.2012.01.036 22385861 PMC3283811

[B43] Shao Q Fan Y Yang L Qin Gao Y From protein denaturant to protectant: comparative molecular dynamics study of alcoholprotein interactions J Chem Phys 2012 136 115101 10.1063/1.3692801 22443795

[B44] Sonnichsen FD Van Eyk JE Hodges RS Sykes BD Effect of trifluoroethanol on protein secondary structure: an NMR and CD study using a synthetic actin peptide Biochemistry 1992 31 8790 8798 10.1021/bi00152a015 1390666

[B45] Nelson JW Kallenbach NR Persistence of the alpha-helix stop signal in the S-peptide in trifluoroethanol solutions Biochemistry 1989 28 5256 5261 10.1021/bi00438a050 2548607

[B46] Padmanabhan S Jimenez MA Rico M Folding propensities of synthetic peptide fragments covering the entire sequence of phage 434 Cro protein Protein Sci 1999 8 1675 1688 10.1110/ps.8.8.1675 10452612 PMC2144428

[B47] Lehrman SR Tuls JL Lund M Peptide alpha-helicity in aqueous trifluoroethanol: correlations with predicted alpha-helicity and the secondary structure of the corresponding regions of bovine growth hormone Biochemistry 1990 29 5590 5596 10.1021/bi00475a025 2386788

[B48] Abergel C Coutard B Byrne D Chenivesse S Claude JB Deregnaucourt C Fricaux T Gianesini-Boutreux C Jeudy S Lebrun R Structural genomics of highly conserved microbial genes of unknown function in search of new antibacterial targets J Struct Funct Genomics 2003 4 141 157 10.1023/A:1026177202925 14649299

[B49] Wu S Skolnick J Zhang Y Ab initio modeling of small proteins by iterative TASSER simulations BMC Biol 2007 5 17 10.1186/1741-7007-5-17 17488521 PMC1878469

[B50] Lin SJ Hu Y Zhu J Woodruff TK Jardetzky TS Structure of betaglycan zona pellucida (ZP)-C domain provides insights into ZP-mediated protein polymerization and TGF-beta binding Proc Natl Acad Sci U S A 2011 108 5232 5236 10.1073/pnas.1010689108 21402931 PMC3069177

[B51] Paisley D Banks S Selfridge J McLennan NF Ritchie AM McEwan C Irvine DS Saunders PT Manson JC Melton DW Male infertility and DNA damage in Doppel knockout and prion protein/Doppel double-knockout mice Am J Pathol 2004 164 2279 2288 10.1016/S0002-9440(10)63784-4 15161660 PMC1615753

[B52] Evans JP Sperm-egg interaction Annu Rev Physiol 2012 74 477 502 10.1146/annurev-physiol-020911-153339 22054237

[B53] Espenes A Harbitz I Skogtvedt S Fuglestveit R Berg KA Dick G Krogenaes A Tranulis MA Dynamic expression of the prion-like protein Doppel in ovine testicular tissue Int J Androl 2006 29 400 408 10.1111/j.1365-2605.2005.00618.x 16390495

[B54] Peoc'h K Serres C Frobert Y Martin C Lehmann S Chasseigneaux S Sazdovitch V Grassi J Jouannet P Launay JM Laplanche JL The human "prion-like" protein Doppel is expressed in both Sertoli cells and spermatozoa J Biol Chem 2002 277 43071 43078 10.1074/jbc.M206357200 12200435

[B55] Rondena M Ceciliani F Comazzi S Pocacqua V Bazzocchi C Luvoni C Chigioni S Paltrinieri S Identification of bovine doppel protein in testis, ovary and ejaculated spermatozoa Theriogenology 2005 63 1195 1206 10.1016/j.theriogenology.2004.06.009 15710203

[B56] Tranulis MA Espenes A Comincini S Skretting G Harbitz I The PrP-like protein Doppel gene in sheep and cattle: cDNA sequence and expression Mamm Genome 2001 12 376 379 10.1007/s003350010285 11331946

[B57] Ecroyd H Sarradin P Dacheux JL Gatti JL Compartmentalization of prion isoforms within the reproductive tract of the ram Biol Reprod 2004 71 993 1001 10.1095/biolreprod.104.029801 15163617

[B58] Gatti JL Metayer S Moudjou M Andreoletti O Lantier F Dacheux JL Sarradin P Prion protein is secreted in soluble forms in the epididymal fluid and proteolytically processed and transported in seminal plasma Biol Reprod 2002 67 393 400 10.1095/biolreprod67.2.393 12135872

[B59] Gatti JL Metayer S Belghazi M Dacheux F Dacheux JL Identification, proteomic profiling, and origin of ram epididymal fluid exosome-like vesicles Biol Reprod 2005 72 1452 1465 10.1095/biolreprod.104.036426 15635128

[B60] Shaked Y Rosenmann H Talmor G Gabizon R A C-terminal-truncated PrP isoform is present in mature sperm J Biol Chem 1999 274 32153 32158 10.1074/jbc.274.45.32153 10542251

[B61] Premzl M Sangiorgio L Strumbo B Marshall Graves JA Simonic T Gready JE Shadoo, a new protein highly conserved from fish to mammals and with similarity to prion protein Gene 2003 314 89 102 14527721 10.1016/s0378-1119(03)00707-8

[B62] Young R Le Guillou S Tilly G Passet B Vilotte M Castille J Beringue V Le Provost F Laude H Vilotte JL Generation of Sprn-regulated reporter mice reveals gonadic spatial expression of the prion-like protein Shadoo in mice Biochem Biophys Res Commun 2011 412 752 756 10.1016/j.bbrc.2011.08.049 21871438

[B63] Baibakov B Boggs NA Yauger B Baibakov G Dean J Human sperm bind to the N-terminal domain of ZP2 in humanized zonae pellucidae in transgenic mice J Cell Biol 2012 197 897 905 10.1083/jcb.201203062 22734000 PMC3384420

[B64] Jovine L Litscher ES Wassarman PM Egg zona pellucida, egg vitelline envelope, and related extracellular glycoproteins Advances in developmental biology and biochemistry 2002 12 31 54

[B65] Gupta SK Bhandari B Shrestha A Biswal BK Palaniappan C Malhotra SS Gupta N Mammalian zona pellucida glycoproteins: structure and function during fertilization Cell Tissue Res 2012 349 665 678 10.1007/s00441-011-1319-y 22298023

[B66] Fernandes I Chanut-Delalande H Ferrer P Latapie Y Waltzer L Affolter M Payre F Plaza S Zona pellucida domain proteins remodel the apical compartment for localized cell shape changes Dev Cell 2010 18 64 76 10.1016/j.devcel.2009.11.009 20152178

[B67] Jovine L Qi H Williams Z Litscher ES Wassarman PM A duplicated motif controls assembly of zona pellucida domain proteins Proc Natl Acad Sci U S A 2004 101 5922 5927 10.1073/pnas.0401600101 15079052 PMC395899

[B68] Ganguly A Bukovsky A Sharma RK Bansal P Bhandari B Gupta SK In humans, zona pellucida glycoprotein-1 binds to spermatozoa and induces acrosomal exocytosis Hum Reprod 2010 25 1643 1656 10.1093/humrep/deq105 20504872

