# Supplementary figures and images for: Inhibition of ovine in vitro fertilization by anti-Prt antibody: hypothetical model for Prt/ZP interaction
Source: Reprod Biol Endocrinol. 2013 Mar 26;11:25. doi: 10.1186/1477-7827-11-25 (PMC3617107; doi:10.1186/1477-7827-11-25)

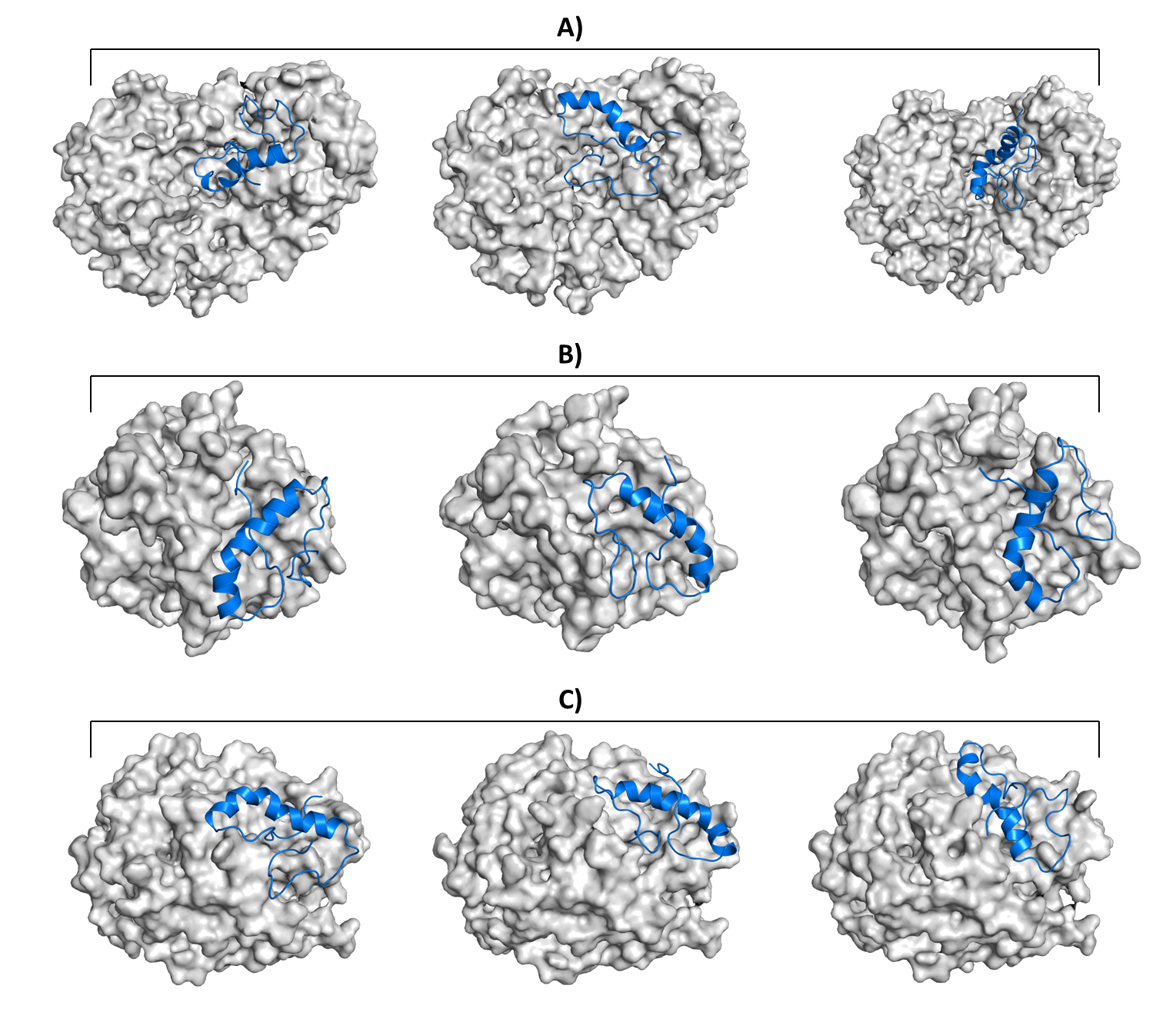

Supplement: Additional file 1 — Figure S1.Images of the three best presenting clusters (Haddock scores), for (A) ZP2, (B) ZP3 and (C) ZP4. [file 1477-7827-11-25-S1.png]
